# Enhancing Reliability and Stability of BLE Mesh Networks: A Multipath Optimized AODV Approach

**DOI:** 10.3390/s24185901

**Published:** 2024-09-11

**Authors:** Muhammad Rizwan Ghori, Tat-Chee Wan, Gian Chand Sodhy, Mohammad Aljaidi, Amna Rizwan, Ali Safaa Sadiq, Omprakash Kaiwartya

**Affiliations:** 1 School of Computer Sciences, Universiti Sains Malaysia, Gelugor 11800, Malaysia; mrizwanghori@student.usm.my (M.R.G.);; 2 Department of Computer Science, Faculty of Information Technology, Zarqa University, Zarqa 13110, Jordan; mjaidi@zu.edu.jo; 3 School of Computing, National University of Computer and Emerging Sciences, Islamabad 44000, Pakistan; i212194@nu.edu.pk; 4 Department of Computer Science, Nottingham Trent University, Clifton Lane, Nottingham NG11 8NS, UK

**Keywords:** Bluetooth Low Energy mesh, optimization, sensors, reliability and stability, multipath AODV

## Abstract

Bluetooth Low Energy (BLE) mesh networks provide flexible and reliable communication among low-power sensor-enabled Internet of Things (IoT) devices, enabling them to communicate in a flexible and robust manner. Nonetheless, the majority of existing BLE-based mesh protocols operate as flooding-based piconet or scatternet overlays on top of existing Bluetooth star topologies. In contrast, the Ad hoc On-Demand Distance Vector (AODV) protocol used primarily in wireless ad hoc networks (WAHNs) is forwarding-based and therefore more efficient, with lower overheads. However, the packet delivery ratio (PDR) and link recovery time for AODV performs worse compared to flooding-based BLE protocols when encountering link disruptions. We propose the Multipath Optimized AODV (M-O-AODV) protocol to address these issues, with improved PDR and link robustness compared with other forwarding-based protocols. In addition, M-O-AODV achieved a PDR of 88%, comparable to the PDR of 92% for flooding-based BLE, unlike protocols such as Reverse-AODV (R-AODV). Also, M-O-AODV was able to perform link recovery within 3700 ms in the case of node failures, compared with other forwarding-based protocols that require 4800 ms to 6000 ms. Consequently, M-O-AODV-based BLE mesh networks are more efficient for wireless sensor-enabled IoT environments.

## 1. Introduction

The Bluetooth Low Energy (BLE) protocol has become increasingly popular for battery-powered sensor-enabled Internet of Things (IoT) devices. The BLE standard was first introduced by the Bluetooth Special Interest Group (SIG) in Bluetooth version 4.0 and has seen further improvements in versions 4.2 and 5. In the initial releases (BLE 4.x), BLE used the traditional Bluetooth Personal Area Network (PAN) paradigm for multi-hop communications and network interconnections. However, BLE 5 has addressed the limitations of previous versions by implementing a pure mesh architecture to support increased network coverage, inter-network connection, and security [1,2,3]. Most BLE-based applications still use a star network architecture and utilize BLE beacons in broadcast mode [1]. Hybrid mesh topologies expand the master–slave piconet concept into interconnected scatternets by combining star and mesh links to enhance the coverage of BLE 4 networks [4,5].

Nevertheless, hybrid mesh networks still face challenges related to reliability and scalability. In contrast, a pure mesh topology overcomes the master–slave limitation by enabling nodes to peer with each other, thereby creating scalable networks. There is currently limited research on the implementation of pure mesh topologies using BLE 5. Additionally, existing BLE-based protocols do not offer routing support for message communication [6,7].

Most BLE-based mesh protocols are designed to work on top of standard Bluetooth star topologies, making use of piconets and scatternets. In traditional BLE mesh networks, there is widespread broadcasting for multi-hop communication, leading to significant overhead and communication delays due to message broadcasting without a routing mechanism.

The AODV (also known as the Ad hoc On-Demand Distance Vector) is a widely used and reliable routing protocol in Wireless Ad Hoc Networks [8]. It functions as a reactive routing protocol and was designed by [9]. The AODV protocol has demonstrated its superiority over other protocols in the same category for wireless ad hoc networks, showing greater efficiency compared to its counterparts [10]. It maintains only necessary routes in the network. AODV is equipped with a routing table that stores the next hop for reaching destinations. Routes will expire if no packets are transmitted along them. Due to its limited knowledge of neighbors, data frame retransmission might experience delays.

In terms of routing-based mesh solutions, only one BLE-based protocol has been developed by [11], which introduces an AODV-based approach for BLE mesh networks. Nonetheless, there are challenges that need to be addressed, such as: (a) high overheads and delays due to the absence of efficient forwarding mechanism; and (b) lack of alternate paths for smooth packet transmission in case of link disruption. In contrast to directed forwarding protocols, flooding techniques are more resilient to link disruption since there is no single point of failure and since an alternate path is always available [12]. Another issue is a low packet delivery ratio (PDR) as compared to mesh protocols. Since mesh utilizes a flooding approach for message replication, it has a higher PDR than directed forwarding. The flooding approach enhances the likelihood of message delivery to the destination nodes but incurs more overheads and delays [13].

To overcome the issues of excessive overhead due to forwarded packets in flooding-based BLE mesh networks, as well as the negative impact on overall PDR due to link disruptions experienced by directed-forwarding protocols such as AODV, additional modifications to AODV-based protocols are required.

Our work enhances the available AODV-based solution for BLE mesh networks to achieve the following:

(i) incorporating multipath forwarding for improving the robustness of the AODV protocol in terms of link setup time, overhead, and link recovery time when dealing with link disruptions;
(ii) improving the resilience of multipath AODV to achieve a PDR comparable to the BLE-based mesh flooding approach using a multipath forwarding mechanism for normal connectivity and when experiencing link disruptions;
(iii) comparing the outcomes of our proposed Multipath Optimized AODV (M-O-AODV) protocol against the performance of the Reverse AODV (R-AODV) protocol [14].

## 2. Related Work

This section discusses the available research related to BLE-based single-path AODV and multipath AODV protocols.

### 2.1. BLE-Based Single Path AODV Protocols

According to the literature, two studies have attempted to limit uncontrolled packet forwarding in order to enhance the BLE mesh protocol.

In [15], we improved the BLE-based AODV protocol developed by [11] in order to minimize the occurrence of unnecessary retransmissions. The optimized AODV (O-AODV) protocol [15] has reduced both channel utilization and the probability of collisions when compared to the AODV protocol.

The weakness of single-path AODV-based forwarding compared to flooding is that node failures result in disruptions to the data forwarding process. In order to overcome this problem, we have investigated the use of multipath enhancements to AODV to study its effectiveness in improving the robustness of the proposed protocol.

### 2.2. Multipath AODV Protocols

This subsection reviews the available literature on multipath AODV for the purpose of designing BLE-based multipath AODV for this research. Note that, other than O-AODV, the protocols presented in Table 1 were designed and evaluated for other wireless technologies. There are no BLE-based implementations of multipath AODV that we are aware of.

An On-demand Multipath Distance Vector (AOMDV) protocol was introduced by [16]. A QoS-MRAODV protocol that is resistant to changes in the dynamic network topology has been suggested in [17]. An Efficient Multipath AODV (EMAODV) protocol was created by [18] in order to regulate the traffic congestion caused by route request (RREQ) rebroadcasting. Ref. [19] suggested an AODV-BR with route backup capabilities. Ref. [20] created a MultiPath AODV (MP-AODV) that incorporates node disjoint routes and the backup route finding procedure during message transmission. A Resilient AODV (RAODV) was presented by [21], which can create many plausible alternate routes and which chooses an alternate route quickly when a path breaks. Ref. [22] developed a robust AODV that includes numerous backup routes, with the highest priority backup route taking over in the event that the primary route fails. Ref. [23] developed the AODV-ABR protocol with the concept of having an alternative route by listening to RREP and data packets. The protocol is adaptive to topology changes. Ref. [24] developed the AODV-Multipath (AODVM) protocol that records the information of duplicate route requests for node disjoint multipath. Ref. [25] presented an AODV with the Guaranteed Bandwidth protocol that included guaranteed bandwidth and backup routing. Ref. [26] developed the Load-Balancing AODV (LBAODV) protocol using the idea of several paths operating simultaneously. A protocol known as AODV Backup Routing with Least Hop count First (LHF) (AODVBRL) was created by [27]. In order to handle numerous homogeneous network interfaces and multipath routing, Ref. [28] suggested the Extended AOMDV for Multi-Interface Multi-Channel (EAOMDV-MIMC) Networks protocol.

Based on the literature described above and summarized in Table 1, it is evident that there have been protocols that use a multipath approach for AODV to improve the performance of the AODV protocol in terms of various performance matrices. Following that, Refs. [14,25] worked on multipath AODV to increase the AODV packet delivery ratio. In addition, Refs. [16,20,25] attempted to increase network performance by reducing end-to-end delay during packet transmission. Ref. [19] created a protocol that uses a mesh-based method to improve network performance in terms of node mobility.

As shown in Table 1, all the protocols have used more or less the same RREQ-RREP pattern, except the M-R-AODV protocol that was authored by [14]. In [14], the authors proposed a reverse route discovery procedure to reduce RREQ-RREP packet loss. According to the authors, legacy AODV protocol may result in disconnection (in the case of instant node failure) as the pre-determined reverse path is used. In M-RAODV, both the source and destination nodes have equivalent roles during the route discovery process, similar to AODV, in terms of sending control messages. Consequently, after receiving the RREQ message from the source node, the destination node floods reverse route requests (R-RREQ) to locate the source node. Upon receiving an R-RREQ message, the source node immediately starts data transmission. In comparison to traditional AODV, the authors claim that this technique has shown better results in cases of disconnection due to node failure along a predefined path.

Recent research on BLE mesh protocols highlights gaps in the enhancement and scalability of BLE mesh protocols. The ability of BLE mesh networks to withstand node failure and mobility is limited, as most BLE mesh topologies are designed for scatternet topologies with connection-oriented communications [4]. In most proposed protocols, the use of a limited number of inter-cluster links hampers the scalability of scatternets.

**Table 1:** Comparison of multipath Ad hoc On-demand Distance Vector (AODV) protocols.

Ref.	Protocol	Protocol Description	PDR	End-to-End Delay	Overhead	Pros (P) and Cons (C)
[15]	O-AODV	Enhanced the BLE-based AODV protocol developed by [11]	82%	5.5 s	26%	P—Given better PDR, End to End Delays, & overheads than BLE based AODV C—node failures result in disruptions to the data forwarding process
[16]	AOMDV	The protocol was designed with many loop-free and link-disjoint pathways in mind	80% to 95%	0.1 to 0.25 s	20% reduction in routing overheads	P—Effective in terms of end-to-end delay C—No detailed analysis of overhead
[17]	QoS-MRAODV	Proposed the concept of a redundant route in which there is one major route and many backup routes	x	0.5 to 1 s	4–5 bits	P—Resistant to dynamic network topology changes C—Lack of packet delivery ratio analysis
[18]	EMAODV	Instead of flooding the entire network, determine a path for route discovery	79% to 80%	0.147 to 0.15 s	x	P—Protocol is capable of controlling the traffic congestion produced by RREQ rebroadcasting; author has proven EMAODV performed better than AODV and DSR C—Lack of network overhead analysis.
[19]	AODV-BR	Protocol is able to listen for RREP from its neighbors for the alternate route table	90% to 95%	15 to 20 ms	x	P—The protocol has the added advantage of having a mesh-based structure and is robust to mobility C—With more pause time protocol not showing, it is better than AODV; no overhead analysis
[20]	MP-AODV	Concepts of backup route discovery process during message transmission and node disjoint routes	90% to 92%	0.0145 to 0.015 s	51 to 53%	P—The protocol provided backup routes and decreased end-to-end delay C—High overheads; not a detailed analysis of performance metrics
[22]	Robust AODV	The system generates several backup routes. If the primary route fails or becomes less favorable, the backup route with the highest priority will take over	x	x	2–5 packets	P—Protocol is resistant to mobility C—No detailed analysis or performance measurements
[23]	AODV-ABL	Concept of having alternative route by listening to RREP and data packets	98% to 99%	0.05 to 0.10 s	Lower than AODV	P—Protocol is adaptive to topology changes C—Proper analysis of measurements is missing
[24]	AODVM	Records the information of duplicate route requests for node disjoint multipath	x	x	x	P—Protocol is robust in the case of node failure C—Author did not given simulation results in terms of PDR, end-to-end delay, overheads, etc.
[25]	AODV-GBR	AODV with Guaranteed Bandwidth protocol having idea of backup routing and guaranteed bandwidth	40% (node moving) 80% (node is static)	50 to 400 ms	x	P—Protocol has shown better data delivery and end-to-end delay C—No overhead analysis
[26]	LBAODV	Concept of simultaneous multiple pathways. In addition, load balancing occurs across several pathways, and energy consumption is dispersed among a large number of nodes	Higher PDR than AODV and AOMDV	x	High overhead as compared to AODV and AOMDV	P—Efficient energy distribution and load balancing C—Better analysis of performance measurements is required
[27]	AODV-BRL	The protocol is based on the idea of enhancing AODV-BR, prioritizing least hop count first (LHF), incorporating backup routing, and expanding Hello and RREP.	Higher PDR than AODV-BR and AODV	Better than AODV-BR but not better than AODV	Lower overheads than AODV-BR and AODV	P—Protocol has the feature of adapting to topology changes C—Performance measurements can be improved
[28]	EAOMDV-MIMC	The concept involves using multipath routing with multiple identical network interfaces, allowing nodes to utilize available communication channels	95%	0–40 ms	10–20%	P—Protocol has given improved performance with many network interfaces on multiple channels C—Detailed analysis of overhead is missing
[14]	M-R-AODV	Ability to establish a number of plausible alternate routes, and when a path breaks, instantly choose an alternate route	76%	0.20 s	x	P—Protocol achieved lower packet loss C—The author did not mention the percentage of packet loss reduction and measurement of other performance metrics

The literature indicates that most protocols employ uncontrolled forwarding for transmitting messages. As a result, routing solutions have demonstrated their superiority in wireless ad hoc networks [8], where they reduce overhead by removing the need for message flooding. Additionally, to address the issue of message flooding, a routing-based approach with multipath support enhances the protocol’s reliability and resilience in the event of a link failure [21].

Based on the discussion and review of the literature previously mentioned, it is essential to have BLE mesh protocols that utilize multiple paths and operate in a connectionless manner to address the limitations outlined. Existing proposed connectionless protocols primarily depend on broadcast-based flooding for packet forwarding. More efficient connectionless protocols, which utilize directional and multipath forwarding, are necessary to minimize the significant packet forwarding overhead inherent in flooding-based solutions (e.g., [29,30]).

## 3. Multipath Optimizations (For Robustness and Better PDR)

In view of the literature discussed in Section 2, one of the weaknesses of the BLE-based protocols that have utilized AODV forwarding compared to flooding is that node failures result in disruptions to the data forwarding process.

In order to overcome this problem, this research investigates the use of multipath enhancements to AODV to study its effectiveness in improving the robustness of the proposed protocol.

Consequently, the AODV algorithm is further enhanced to the Multipath Optimized AODV (M-O-AODV) version in which duplicate route request packets are not discarded at the destination node but rather processed as normal route request packets by increasing the number of route replies generated by the route reply function.

In M-O-AODV, RREQ transmission from source to destination creates multiple reverse paths at destination nodes. At the source and intermediate nodes, multiple RREPs traverse these reverse paths to form multiple forward paths to the destination. Although multiple alternate paths can be used, the proposed M-O-AODV investigates the use of only one alternate path, as a compromise between increasing robustness and minimizing extra overhead.

Moreover, M-O-AODV uses the forwarding data available in the underlying AODV protocol to curb the RREQ forwarding overhead that will be incurred due to the multipath feature.

Multipath AODV has two conflicting objectives. While the TTL value should be kept low to avoid excessive forwarding of RREQ packets, we also need to discover alternative paths in addition to the initial shortest path identified by the first RREP packet. Hence, RREQ forwarding in downstream forwarding nodes not in the RREP shortest path should not be suppressed immediately.

M-O-AODV addresses this by means of the RREQ retries mechanism. A downstream forwarding node not currently on the forwarding path will reset the TTL of a received RREQ packet with zero TTL and continue forwarding it as long as the RREQ retries count is non-zero.

However, it will stop forwarding RREQ packets, stopping the expanding ring search, after it has received an RREQRECV message from the destination with the given source sequence number. By means of the RREQRECV mechanism, we can tailor the number of supported alternative paths dynamically for future scalability.

In the current implementation of M-O-AODV, the number of alternative paths is set to two. Therefore, once two route requests are processed to form alternate paths, the destination node will check if the RREQRECV message has been sent to inform the downstream forwarding nodes not in the identified paths to stop forwarding RREQ messages with that sequence number. If none were sent previously, the destination node will broadcast the RREQRECV packet with its TTL equal to the hop count for the given RREQ to its neighbors.

To support effective multipath forwarding in the BLE network, we assume that the node density ρ= n/A [2] is high enough that each node would have at least two neighbors, which ensures that different paths from source to destination can be established. For the proposed M-O-AODV, we calculated the area of coverage A for a given number of nodes placed randomly with uniform distribution using Equation (1) [2], given that the probability that the network is k-connected is 100%, k = 2, and transmission range $r_{0}$ = 10 m. We then used Equation (2) in [3] to calculate the worst-case number of hops (H) for the given area of coverage A. Additionally, the formula for the TTL is provided in Equation (3), given as a function of the number of nodes n, which is expected to increase in proportion to the worst-case number of hops.

**Figure 1:**
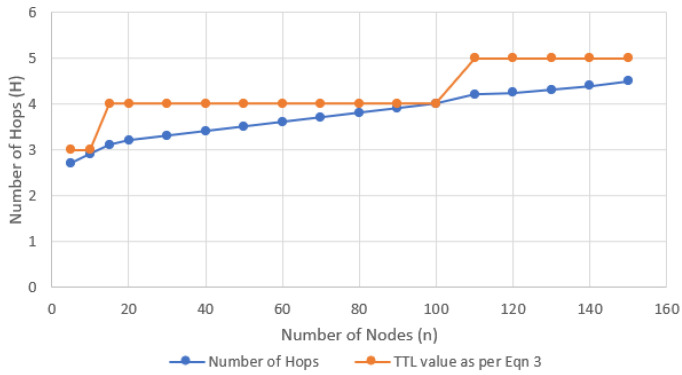
Scalability of M-O-AODV in terms of the TTL and worst-case number of hops for a given number of nodes (with k-connectivity, where k = 2 and r_0 = 10 m).

Figure 1 presents an analysis of the number of hops and TTL versus network size, based on the assumption that k = 2 will result in at least two disjoint paths between the source and destination nodes. By using a lower initial value of TTL, M-O-AODV reduces excessive flooding of RREQ packets through the network, while the RREQ retries mechanism increases the reachability of RREQ packets to a maximum of 3xTTL hops. Moreover, the protocol will stop RREQ forwarding after the current TTL expires when the destination sends RREQRECV packets to the forwarding nodes. This modification has not been adopted by other multicast AODV protocols studied.

In addition, the area of coverage A calculated using Equation (1) is shown in Table 2, illustrating the scalability of the proposed approach for typical indoor sensor-enabled IoT applications, aligned with the authors’ findings in [31].

**Table 2:** Number of nodes in the network (n) vs. area coverage (A).

No. of Nodes in the Network (n)	Area Coverage (A) m^2^
5	213
10	368
15	550
20	718
30	1029
40	1330
50	1625
60	1913
70	2198
80	2479
90	2757
100	3032
110	3305
120	3576
130	3844
140	4111
150	4377

(1) $$P\left(G\;\mathrm{is}\;k\text{-}\mathrm{connected}\right)=(1-\sum_{N=0}^{k-1}\frac{{\left(\rho .\pi .r_{0}^{2}\right)}^{N}}{N!}.e^{-\rho .\pi .r_{0}^{2}}{)}^{n}$$

(2) $$H=\sqrt{\frac{n}{\pi .\rho .r_{0}^{2}}}$$

(3) TTL=⌈log(n)+2⌉

where, for Equations (1)–(3) [2,3]

P = probability G is k-connected
n = number of nodes in the network
k = 2 (assumption that at least two links exist between the nodes)
A = area of coverage
ρ = n/A
$r_{0}$ = transmission range of each node
H = number of hops

**Figure 2:**
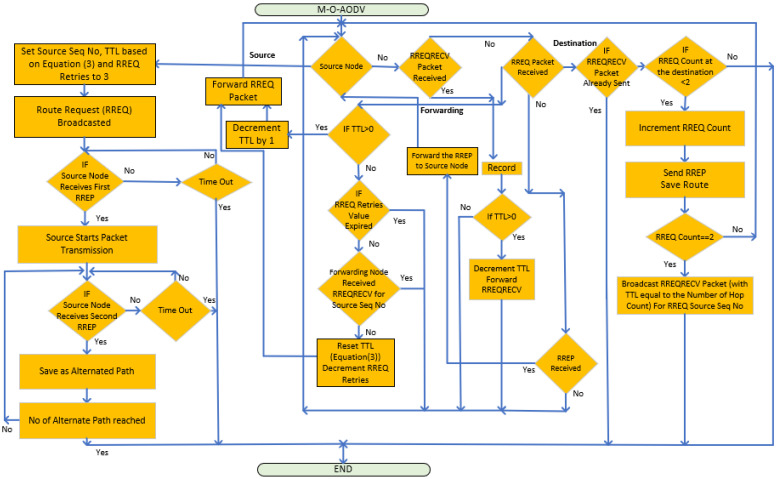
M-O-AODV protocol message flow chart.

Moreover, the flowchart in Figure 2 shows the data flow for the M-O-AODV protocol.

## 4. Proposed M-O-AODV Implementation Details

This section discusses the implementation details related to the proposed M-O-AODV protocol.

### 4.1. Operating System

The proposed protocol has been developed using the Zephyr operating system (OS), which is specifically tailored for resource-limited embedded devices and has a minimal footprint. This OS, in which the proposed protocol is written, is built on a kernel with a tiny footprint to cater to resource-limited embedded devices. These devices can vary from basic environmental sensors and LED wristbands to sophisticated sensor-enabled IoT applications and comprehensive integrated controllers.

### 4.2. Testbed Design and Topology

**Figure 3:**
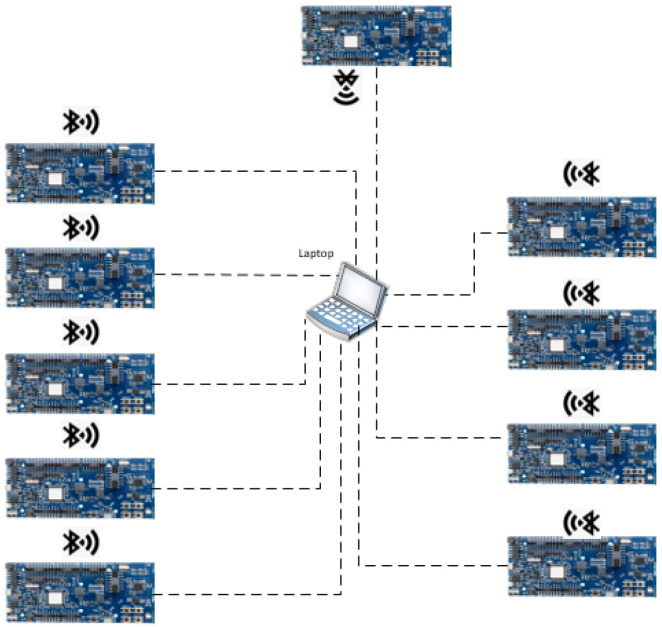
Display of the testbed setup featuring USB connections utilized for experiment configuration and data collection.

**Figure 4:**
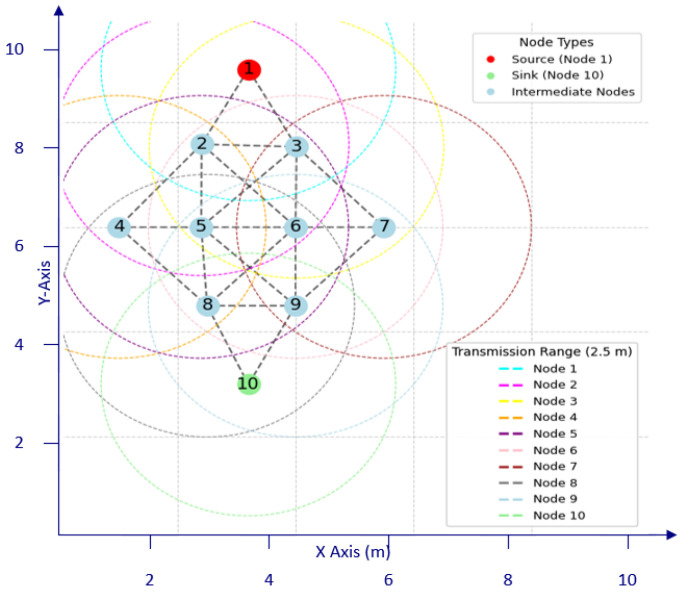
Multipath topology with 10 nodes.

The experimental testbed comprises 10 nRF52840DK development kits, Nordic Semiconductor, Trondheim, Norway, as depicted in Figure 3, which are programmed with the proposed protocol. Each development kit is connected to a laptop via a serial port using a USB hub in order to record experimental data. The testbed was designed to validate the M-O-AODV protocol and to measure its performance in an unobstructed open environment, where the nodes were placed on 1 m high wooden tables within the test area. Moreover, the topology used in the experiments is shown in Figure 4, where a single source transmits data to the sink through four hops within a partial mesh network, providing multiple possible routes. Figure 4 also illustrates transmission range circles in different colors, as indicated by the legend, representing the transmission range of each node. In this configuration, nodes are arranged such that only those within each other’s transmission range are connected, while nodes outside of this range remain unconnected.

To evaluate the proposed M-O-AODV protocol, it is tested using the partial mesh topology as used by [15] to assess the proposed protocol’s efficiency and robustness.

### 4.3. Performance Measurement Metrics Used for Experiments

For a better understanding of the experimental results, the performance measurement metrics used for the experiments are explained in this section.

#### 4.3.1. Packet Delivery Ratio (PDR)

When the packet delivery ratios are high, overall performance improves. It is calculated as follows:

(4) $$\mathrm{PDR}=\frac{\sum \mathrm{Packets}\;\mathrm{received}\;\mathrm{by}\;\mathrm{all}\;\mathrm{destination}\;\mathrm{nodes}}{\sum \mathrm{Number}\;\mathrm{of}\;\mathrm{hops}\;\mathrm{from}\;\mathrm{the}\;\mathrm{destination}}.$$

#### 4.3.2. RREQ-RREP Setup Time

This is the overall time taken by the protocol from the moment the route request is initiated until it reaches the destination, and from when the destination sends the route reply message to the source and the source receives it. This duration is calculated as follows:

(5) RREQ−RREP SetupTime=t_rreq_received−t_rrep_sent.

#### 4.3.3. Link Reestablishment Time (LRET) Measurement

We have calculated the LRET as the time difference between the last packet received before the link was switched off and the first packet received after the link was reestablished:

(6) LRET=t_first_pkt_recv_after_link_reestb − t_last_pkt_rec_before_link_off.

#### 4.3.4. Overhead

The overhead is calculated for this research as follows:

(7) $$\mathrm{Overhead}=\frac{\mathrm{Total}\;\mathrm{No}.\;\mathrm{of}\;\mathrm{Control}\;\mathrm{Packets}\;(\mathrm{RREQ}\;\&\;\mathrm{RREP})}{\mathrm{Total}\;\mathrm{Packets}\;\mathrm{Received}}.$$

### 4.4. Common Experiment Setup and Configurations

This research involved conducting experiments using a partial mesh multipath topology, as illustrated in Figure 4. Furthermore, two different scenarios were tested 10 times to ensure accurate results, with 100 packets sent from the source using the lowest transmit power to keep the testbed manageable. Each scenario consisted of a single source and a single sink. The general experiment setup and configurations are outlined in Table 3.

## 5. Experimental Results

The results of the experiments performed on the proposed M-O-AODV protocol and on one of the available multipath R-AODV protocols are discussed in this section.

### 5.1. Experiments—Scenario 1: Comparison of M-O-AODV with Available BLE-Based Protocols and R-AODV in Terms of Various Performance Metrics

This scenario is created to evaluate the proposed M-O-AODV protocol’s RREQ-RREP setup time, overhead, and PDR in comparison to O-AODV, AODV, R-AODV, and mesh protocols.

**Figure 5:**
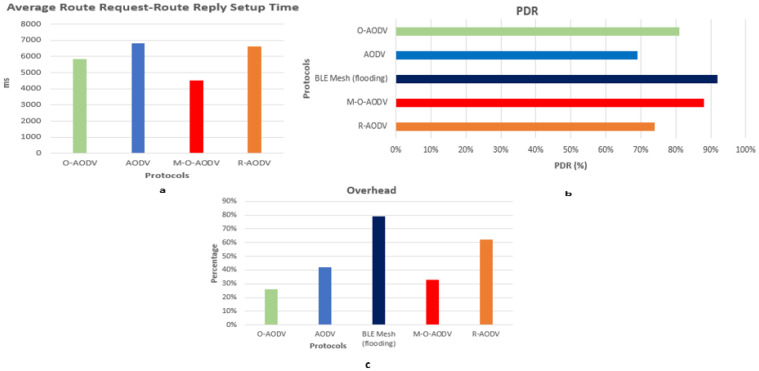
Results of the experiments performed on the proposed M-O-AODV protocol for measuring RREQ-RREP Setup Time, PDR and Overhead.

As shown in Figure 5a, the M-O-AODV protocol’s route request to route reply setup time is 4500 milliseconds, which is better than the O-AODV, AODV, and R-AODV protocols, which take 5800, 6800, and 6700 milliseconds, respectively. Also, M-O-AODV has shown a better PDR of 88%, as shown in Figure 5b, compared to AODV (78%), O-AODV (81%), and R-AODV (74%), but is slightly less than the mesh protocol, which has a PDR of 92% due to the nature of uncontrolled message forwarding in the mesh topology. Figure 5c illustrates that the overhead of M-O-AODV and O-AODV, at 33% and 27%, respectively, is less than AODV, R-AODV, and mesh, at 41%, 61%, and 79%, respectively.

### 5.2. Experiments—Scenario 2: Comparison of M-O-AODV with Available BLE-Based Protocols and R-AODV for Robustness

In this scenario, robustness of the proposed M-O-AODV protocol (in the event of a link failure) in comparison to other protocols is tested. For this scenario, a number of nodes placed manually in the testbed to form a partial mesh topology with the indicated node-to-node links (Figure 4) has been utilized, in which a single source sends data to the sink through the mesh. Based on Equation (3) and the size of the testbed used for our experiments, the TTL was set to 3 as a configuration parameter. The LRET is used to determine the robustness of M-O-AODV with respect to link disruptions, where lower LRETs indicate a shorter link disruption duration and hence faster recovery (better robustness) of a given protocol. For this scenario, once the experiment has reached steady-state transmission between source and destination, a selected forwarding node in the active path is disabled, and the LRET measured. This process is repeated for other forwarding nodes in the path in order to calculate the average link reestablishment time for the given path. Furthermore, experiments have been conducted to analyze the effects on LRET and instantaneous PDR in the event of link failure.

This section presents the results obtained regarding robustness of the proposed protocol in the case of link disruption or node failure. The section is divided into two subsections; the first one discusses the experiments depicting the link reestablishment time in the case of a node failure, and the second one demonstrates the effects on instantaneous PDR values in the case of a node failure, in order to depict the robustness of the proposed protocol. It is highlighted that the node placement in Figure 4 was set up based on manual configuration to create the required topology (node-to-node links). This configuration was used in the experimental testbed. However, for measuring the statistics for the probability of end-to-end hops, the calculations for k-connectivity assume a random uniform distribution.

#### 5.2.1. Experiment on Link Reestablishment Time in the Case of Node Failure

**Figure 6:**
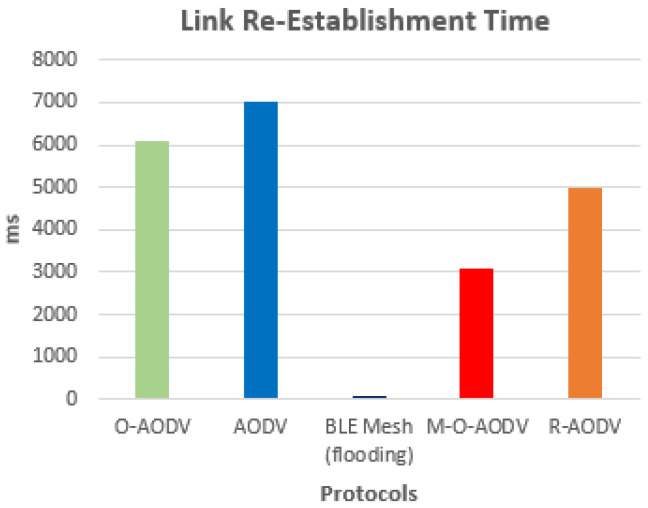
Link reestablishment time.

Figure 6 illustrates the time needed to restore a connection when multiple paths are available, which is significantly less than the time needed to reestablish a connection when only one path is available.

#### 5.2.2. Effects on PDR in the Case of Link Disruption/Node Failure

This experiment compares the effects of link disruption on PDR for the proposed protocol M-O-AODV in addition to that of the O-AODV, AODV, R-AODV, and mesh protocols. As shown in Table 4, when comparing the O-AODV, AODV, and R-AODV protocols, M-O-AODV has the least effect on PDR after the link is reestablished. Consequently, link disruption does not affect mesh protocol due to its uncontrolled forwarding feature and provides a linear PDR. Table 4 also depicts the effects on PDR in the case of link disruption. As per the results, M-O-AODV and mesh protocols have shown robustness against link disruption due to the availability of alternate paths.

## 6. Discussion

M-O-AODV has been evaluated in actual testbed scenarios, unlike any other multipath AODV protocols to our knowledge. The M-O-AODV protocol showed superior performance across different metrics, demonstrating improvements in overhead compared to O-AODV and flooding-based mesh protocol. Additionally, the overhead of the proposed protocol was found to be lower than that of the protocols discussed in references [12,16]. In addition, M-O-AODV also shows better RREQ-RREP setup time in comparison with O-AODV.

Consequently, as a result of the availability of multipath, the M-O-AODV protocol with multipath has provided a better PDR of 88% compared to protocols that follow single-path. So, the PDR performance has been boosted through further optimization of the O-AODV protocol to support multipath, which has reduced the 10% difference in PDR performance to 4%, making it comparable to the mesh protocol performance.

The protocols mentioned in references [19,20] failed to deliver satisfactory results in terms of PDRs and overhead. However, the methodology outlined in reference [21] yielded similar findings, with a PDR of 90% and an overhead of 30%. However, the protocol has not been validated in actual conditions. Moreover, compared to the protocols discussed in references [10,11,18,22], M-O-AODV demonstrated higher PDR, and the authors of those protocols did not assess the overhead, which is crucial for ad hoc networks.

When multiple paths are available, the setup time required to reestablish a connection is significantly less (i.e., 4200 ms with multipath O-AODV) than when only one path is available. Moreover, as the results show, the time required to re-establish a connection when multiple paths are available is significantly less than the time required when only one path is available. This is due to the fact that the protocol following single-path has to pass through the process of route request to route reply setup in the case of node failure or link disruption, which incurs extra time as compared to the protocol having multiple paths. Consequently, the M-O-AODV protocol has ensured a faster route recovery due to an alternate path in the routing table that has been utilized in the event of a node failure.

However, the mesh protocol has demonstrated extremely negligible or no link disruptions in the event of a link failure, as mesh utilizes uncontrolled broadcasting/forwarding, which enables data to travel through all possible paths and reach the destination. However, this forwarding ability incurs a high overhead. Additionally, in the flooding-based mesh protocol, duplicated forwarded packets contribute heavily to the average end-to-end delay [32].

Instantaneous PDR measurements in the case of link disruption have proven that all the protocols have given a decreased PDR immediately after the link has been reestablished. Just like mesh, M-O-AODV has shown a linear increase and decrease in PDR as compared to the other protocols. Subsequently, M-O-AODV has shown a better average instantaneous PDR of 88% as compared to the O-AODV, R-AODV, and AODV protocols. However, mesh has given better PDR as compared to the M-O-AODV protocol due to the fact that mesh follows connectionless uncontrolled forwarding, and the link disruption does not have much impact on its PDR. In view of this, M-O-AODV, being a controlled forwarding protocol, has proven its robustness against link disruption by giving better instantaneous PDR values as compared to single-path protocols. Also, all the protocols have given lower instantaneous PDR immediately after link re-establishment. However, M-O-AODV recovers faster from link disruption and gives better PDR when the link is reestablished. Additionally, the overhead of the proposed protocol was found to be lower than that of the protocols discussed in references [12,16].

Based on [32], directed forwarding protocols such as M-O-AODV have an advantage over flooding-based BLE mesh protocols, since flooding in BLE mesh topologies results in significant network delays due to excessive link contention. This becomes progressively more significant as the number of full-function nodes involved in multi-hop packet forwarding increases.

This study implements the R-AODV protocol proposed by [14] to compare performance with the proposed M-O-AODV protocol. M-O-AODV performed better than the R-AODV protocol in terms of overhead, PDR, and robustness against link disruptions via multipath support. The reason for M-O-AODV’s superior performance is that it transmits a smaller number of control messages, whereas R-AODV transmits a larger number of control messages because it employs the reverse route request methodology for route reply messages, resulting in higher overhead with less PDR.

Furthermore, for M-O-AODV, the multipath feature has been incorporated in the O-AODV to boost the robustness of the proposed protocol in the event of a link failure, which successfully decreased the LRET and enhanced the PDR, and which has further improved the performance of the proposed protocol beyond what was achieved by R-AODV.

M-O-AODV assumes the use of existing security mechanisms provided by the BLE mesh protocol to provide authentication, packet encryption, and data integrity.

Consequently, the contributions of this paper include optimizing multipath AODV algorithms to reduce the overhead of the route discovery process, as well as controlling the increase in overhead due to RREQ forwarding when the network size increases. This is achieved by using a lower initial value of the time-to-live (TTL) and a mechanism to stop forwarding of the RREQ packets when paths have been determined (by means of the RREQRECV and the RREQ retries mechanism). We believe that this is unique to our proposed protocol M-O-AODV.

The limitations of the proposed protocol are that the PDR can still be affected by link disruptions compared to a flooding-based BLE mesh approach. To overcome this issue, additional paths may be provisioned at the cost of increased overheads. Alternatively, increasing the node density to ensure that more neighbors are in range of each forwarding node can also improve the k-connectivity of the overall network.

## 7. Conclusions

M-O-AODV has demonstrated its robustness by giving better average PDR as compared to the other multipath and single-path protocols. Subsequently, decreased link reestablishment time (LRET) values in the case of node failure have proven that the proposed protocol could recover faster even in the case of link breakage.

Furthermore, the lower overheads for M-O-AODV will benefit the network by putting less load on it. In addition, the multi-path support feature in the proposed M-O-AODV protocol has given comparable PDR compared to the mesh protocols for improved reliability and network scalability. Consequently, the proposed protocol should be much more scalable compared to existing flooding-based mesh protocols used by BLE.

In this paper, we have employed the default security features the BLE mesh protocol provides. There is, however, significant potential for future work to enhance our proposed protocol by integrating additional security features against security threats such as denial of service and replay attacks, thereby strengthening its security while maintaining its efficiency.

In future research, M-O-AODV should be tested on mobile nodes to validate its efficacy further. Also, there is a need to develop protocols for BLE mesh networks that provide effective multicast data transmission. Furthermore, efficient auto-configuration procedures are required to facilitate the bootstrapping of BLE pure mesh networks. There is also a need to analyze the performance of BLE mesh protocols with energy efficiency as a primary focus.

## Figures and Tables

**Table 3:** Common experiment setup and configurations.

Experimental Parameters	
No. of Experiments	10
Link Speed	2 Mbps
Data Rate	10 PDUs per second
Packet Size	15 bytes
Number of Packets Sent from the Source	100
Sources & Sinks	1 source, 1 sink
Transmission Power	−40 dBm
Transmission Range (r0)	Approximately 2–2.5 m
Relaying	Enabled
Provisioning	Enabled with hardcoded values
Packet Duplication Rate	3 transmissions with a 20 ms interval
TTL	Set based on number of nodes and Equation (3) TTL =3 for the experiments

**Table 4:** Effects on PDR in the case of link disruption.

Protocols	Link Reestablishment Time (s)	Time (s) to Reach Max PDR	Instantaneous PDR (%) after Link Reestablishment	Average PDR (%)
O-AODV	5	8	66%	83%
AODV	7	11	52%	70%
BLE Mesh (flooding)	Not Applicable	Not Applicable	95%	95%
M-O-AODV	3	2	88%	88%
R-AODV	6	10	67%	74%

## Data Availability

All related data will be provided on request to corresponding authors for research purpose only.

## List of Abbreviations

This manuscript uses the following abbreviations:

AODV	Ad hoc On-demand Distance Vector
BLE	Bluetooth Low-Energy
IoT	Internet of Things
LRET	Link Reestablishment Time
ms	Millisecond
M-O-AODV	Multipath Optimized AODV
O-AODV	Optimized AODV
OS	Operating System
PAN	Personal Area Network
PDR	Packet Delivery Ratio
RTOS	Real-Time Operating System
R-AODV	Reverse AODV
RREQ	Route Request
RREP	Route Reply
R-RREQ	Reverse Route Request
R-RREP	Reverse Route Reply
SIG	Special Interest Group
TTL	Time-To-Live
WAHN	Wireless Ad Hoc Network

## List of Symbols

This manuscript uses the following symbols:

∑	Summation sign
%	Percentage
=	Equals symbol
/	Division symbol
−	Subtraction (if used with a mathematical equation or formula)
*D*	Average end-to-end delay or average one-way delay
*i*	Packet identifier
Mbps	Megabits per second
ms	Millisecond
*n*	Number of packets successfully delivered
Tr	Reception time
Ts	Send time
t_rreq_received	Time route request received
t_rrep_sent	Time route reply sent
t_status_sending_resumed	Time status sending resumed
t_last_status_sent	Time last status sent
ρ	n/A
n/A	n = Number of nodes in the network, A = Area of coverage
$r_{0}$	Transmission range of each node
